# Presence and Variability of the Microbiome in Perivascular Adipose Tissue: A Whole-Genome Sequencing Study in Dahl SS Rats

**DOI:** 10.3390/life16040609

**Published:** 2026-04-07

**Authors:** Sameera Mahimkar, Janice M. Thompson, Christopher B. Blackwood, Stephanie W. Watts, Carolina B. Restini

**Affiliations:** 1 Department of Pharmacology and Toxicology, College of Osteopathic Medicine, Michigan State University, East Lansing, MI 48824, USA; mahimka1@msu.edu (S.M.); janthomp@msu.edu (J.M.T.); wattss@msu.edu (S.W.W.); 2 Department of Plant, Soil, and Microbial Sciences, Michigan State University, East Lansing, MI 48824, USA; blackw79@msu.edu; 3 Department of Plant Biology, Michigan State University, East Lansing, MI 48824, USA

**Keywords:** microbiome, perivascular adipose tissue, immune cells, whole-genome sequencing, vascular biology, Dahl SS rats

## Abstract

**Background**: Perivascular adipose tissue (PVAT) contains adipocytes and a stromal-vascular fraction with immune cells that modulate the adjacent vasculature. The presence of immune cells in PVAT of vascular beds is poorly understood—are they resident or recruited? We propose a novel resident microbiome present in PVAT, given the immune-rich stromal environment. **Hypothesis**: We hypothesized the existence of distinct bacterial and viral communities in healthy PVAT compared to non-PVAT adipose tissues. **Methods**: PVAT samples from thoracic and abdominal aorta, mesenteric resistance arteries, non-PVAT tissues (subscapular brown adipose tissue, retroperitoneal white adipose tissue), and fecal samples were collected one year apart from male Dahl SS rats, split into two cohorts (2023 and 2024, *n* = 3 each). Whole-genome shotgun sequencing (CosmosID) and 16S rRNA gene analysis assessed microbial relative abundance. **Results**: PVAT harbored bacterial and viral sequences, and species composition varied significantly between cohorts. Bacterial and viral fecal samples showed lower variability. **Conclusions**: PVAT microbiome differed dramatically from the fecal microbiome, with temporal influences on bacterial and viral diversity, marking the first such report. Despite inherent limitations, these findings establish the potential of PVAT microbiota in vascular biology and immune modulation, paving the development of microbiome-targeted drugs to address vascular dysfunctions.

## 1. Introduction

Perivascular adipose tissue (PVAT) [1,2,3,4] surrounds blood vessels such as the aorta [5,6,7], renal arteries [8], coronary arteries [9], and mesenteric arteries [10], and plays a key role in regulating vascular function through paracrine effects on vascular tone, inflammation, and remodeling [11,12,13,14]. Structurally, PVAT consists of adipocytes and a stromal vascular fraction (SVF) enriched with immune cells, including macrophages, T lymphocytes, and dendritic cells, which coexist under physiological conditions [15]. These immune populations contribute to vascular homeostasis or pathology depending on context and may serve as an interface between metabolic and immune processes [11,16,17,18,19]. However, key aspects of PVAT biology remain unclear, including the origin and regulation of immune cell populations, as well as whether PVAT composition varies across vascular beds or differs from that of non-PVAT adipose depots.

An emerging area of interest is the role of the microbiome—including bacteria, viruses, and fungi—in shaping tissue-specific immune environments. Microbial populations have been identified in human adipose tissues, including subcutaneous and visceral depots [20,21], and the gut microbiota is a well-established regulator of host immunity and inflammation [22,23,24]. However, the existence and composition of a microbiome within PVAT remain poorly understood. Given the immune-rich nature of PVAT, it is plausible that microbial signatures may contribute to its immunomodulatory functions, with potential implications for vascular health and disease.

The Dahl salt-sensitive (SS) rat is a well-established model of cardiovascular dysfunction and adipose tissue dysregulation [16,25,26,27], providing a relevant system to investigate PVAT-associated microbiome. In this study, we used whole-genome sequencing (WGS) to characterize bacterial and viral profiles in PVAT from the aortic and mesenteric vascular beds and compared them with those in non-PVAT adipose tissues. We hypothesized that microbial sequences are detectable in PVAT and may differ across vascular beds and relative to non-PVAT depots. This work aims to provide initial insights into the presence and variability of microbial signals in PVAT and to inform future investigations into their potential biological relevance.

## 2. Methods

### 2.1. Animal and Sample Collection

#### 2.1.1. Animals

All animal procedures were approved by the Michigan State University Institutional Animal Care and Use Committee (IACUC; PROTO202200001) and adhered to the *Guide for the Care and Use of Laboratory Animals* [28] (8th edition, 2011) and ARRIVE guidelines [29].

Male Dahl SS rats were obtained from Charles River Laboratories (Indianapolis, IN, USA). A total of six rats (*n* = 6), aged 4–5 weeks at the time of arrival, were included in this study. Rats were housed under standard laboratory conditions with *ad libitum* access to water. They were fed the same standard diet (Teklad, West Lafayette, IN, USA), composed of 18.6% protein, 6.2% fat, 3.5% crude fiber, and 14.7% insoluble fiber.

Rats 1–3 were euthanized in 2023, while rats 4–6 were euthanized in 2024. Rat 1, singly housed, was euthanized 9 January 2023; weight at euthanasia: 450 g. Rats 2 & 3 (housed together in one cage) were euthanized on 4 April 2023, weighing 402 g and 410 g, respectively. Rats 4, 5 & Rat 6 (housed together in one cage) were euthanized on 23 February 2024, weighing 421 g, 431 g, and 408 g, respectively. For tissue isolation, rats were euthanized with sodium pentobarbital (60–80 mg/kg, IP).

#### 2.1.2. Tissue Collection

To minimize contamination risks inherent to low-biomass microbiome studies, all sample collection and processing steps were performed under strict aseptic conditions, including dissection in a disinfected laminar flow hood, use of autoclaved instruments, and sterile, single-use consumables. The detailed protocol is described in the Supplementary Materials (File S1 Tissue Collection).

The abdomen of the rat was swabbed with ethanol. Using sterilized surgical instruments, the following tissues were harvested: thoracic aortic perivascular adipose tissue (taPVAT), abdominal aortic PVAT (aaPVAT), mesenteric PVAT (mesPVAT), subscapular brown adipose tissue (BAT), retroperitoneal white adipose tissue (WAT), and feces. The fecal pellet was removed by expressing the pellet from the rectum directly into a sterile tube. The fecal samples served as a positive control for the microbiome. Arterial PVATs were placed in physiological salt solution (PSS [mM]: NaCl 130; KCl 4.7; KH_2_PO_4_ 1.18; MgSO_4_·7H_2_O 1.17; NaHCO_3_ 14.8; dextrose 5.5; CaNa_2_EDTA 0.03, CaCl_2_ 1.6 [pH 7.2]) for rapid dissection from the artery and placed into a sterile microcentrifuge tube.

**Figure 1:**
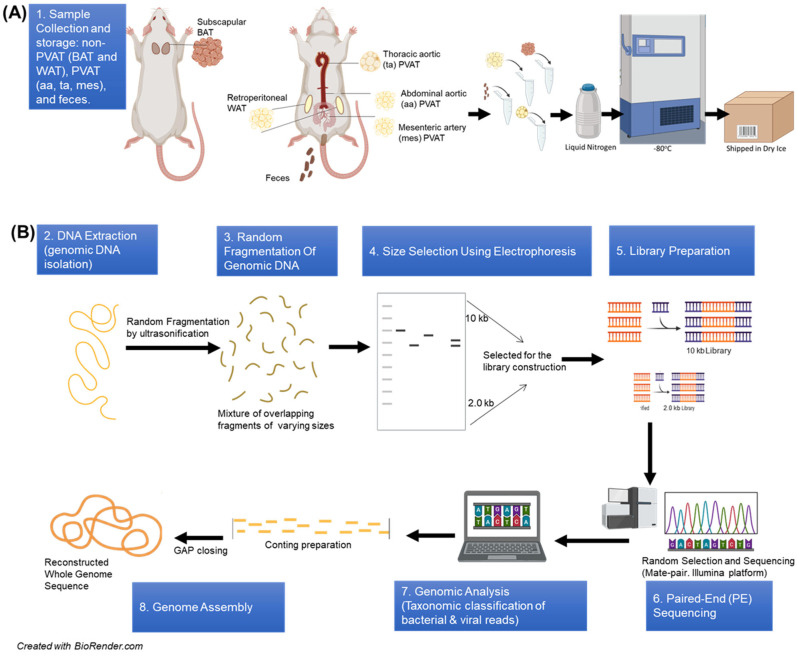
Schematic representation of the whole-genome shotgun sequencing (WGS) laboratory and bioinformatics flow. (**A**) 1. Sample collection and storage: non-PVAT (BAT: brown adipose tissue, WAT: white adipose tissue), PVAT (aa: abdominal aorta, ta: thoracic aorta, mes: mesenteric), and feces. (**B**) DNA sequencing, all microbial DNA in the sample is fragmented into small pieces for next-generation sequencing: 2. isolation of genomic DNA, 3. random fragmentation of genomic DNA, 4. size selection using electrophoresis, 5. library construction, 6. paired-end sequencing (PE sequencing), 7. genomic analysis, 8. genome assembly (CosmosID Metagenomics Cloud, app.cosmosid.com, CosmosID Inc. www.cosmosid.com).

All samples were immediately snap-frozen in liquid nitrogen and stored at –80 °C until DNA extraction (Figure 1A). Samples were subsequently shipped on dry ice to CosmosID^®^ for microbiome profiling via whole-genome shotgun sequencing. Samples were received by CosmosID with sufficient remaining dry ice, and samples were run blinded.

### 2.2. DNA Extraction and Whole-Genome Sequencing

DNA extraction was performed by CosmosID^®^ using proprietary protocols optimized for high-yield, high-quality microbial DNA isolation from low-biomass tissue and fecal samples [30]. Tissue samples were homogenized and subjected to the processes described below and depicted in Figure 1B. When non-proprietary in nature, protocol information was shared by CosmosID^®^ as shown here.

#### 2.2.1. DNA Extraction

DNA from samples was isolated using the QIAGEN (Germantown, MD, USA) DNeasy PowerSoil Pro Kit, according to the manufacturer’s protocol. Extracted DNA samples were quantified using Qubit 4 fluorometer and Qubit™ dsDNA HS Assay Kit (Thermofisher Scientific, Waltham, MA, USA).

#### 2.2.2. Library Preparation

DNA libraries were prepared using the Nextera XT DNA Library Preparation Kit (Illumina, San Diego, CA, USA) and Nextera Index Kit (Illumina, San Diego, CA, USA) with total DNA input of 1 ng. Genomic DNA was fragmented using a proportional amount of Illumina Nextera XT fragmentation enzyme (San Diego, CA, USA). Combinatory dual indexes were added to each sample followed by 12 cycles of PCR to construct libraries. DNA libraries were purified using AMpure magnetic Beads (Beckman Coulter, Brea, CA, USA) and eluted in QIAGEN EB buffer (Germantown, MD, USA). DNA libraries were quantified using Qubit fluorometer and Qubit™ dsDNA HS Assay Kit (Thermofisher Scientific, Waltham, MA, USA). Libraries were then sequenced on an Illumina NovaSeq X platform at 2 × 150 bp (Illumina, San Diego, CA, USA). Appropriate negative (nuclease-free, molecular-grade water) and positive (Zymobiomics, Zymo Research, Orange, CA, USA) controls were run internally for quality assurance and contamination monitoring throughout DNA extraction, library preparation, and sequencing. Library preparation incorporated unique dual indexing to reduce index cross-contamination.

#### 2.2.3. Taxonomic Profiling Methods

The system uses a high-performance k-mer data-mining algorithm that rapidly disambiguates millions of short sequence reads into discrete genomes that generate the observed sequences. The pipeline has two separable comparators: the first consists of a pre-computation phase for reference databases, and the second is a per-sample computation (CosmosID Metagenomics Cloud, app.cosmosid.com, CosmosID Inc. www.cosmosid.com). The input to the pre-computation phase is a database of >150,000 Genome Taxonomy Database-based, that is, databases of reference genomes, virulence markers, and antimicrobial resistance markers that CosmosID scientists continuously curate. The output of the pre-computational phase is a phylogenetic tree of microbes, together with sets of variable-length k-mer fingerprints (biomarkers) that are uniquely associated with the tree’s branches and leaves. The second per-sample computational phase searches the hundreds of millions of short sequence reads, or alternatively, contigs from draft de novo assemblies, against the fingerprint sets. This query enables the sensitive yet highly precise detection and taxonomic classification of microbial Next-Generation Sequencing (NGS) reads. The resulting statistics are analyzed to return the fine-grain taxonomic and relative abundance estimates for microbial NGS datasets. To exclude false-positive identifications, the results are filtered using a threshold derived from internal statistical scores computed from a large set of diverse metagenomes. The same approach is used to enable sensitive and accurate detection of genetic markers associated with virulence and antibiotic resistance. Complete sequencing depth data is provided in the Supplementary Materials (File S2).

### 2.3. Data Analysis

All six male Dahl salt-sensitive rats (rats 1–6) were initially included in the analysis. Microbiome community composition was assessed using percent relative abundance at the species level, calculated separately for bacteria and viruses, based on data generated from whole-genome shotgun sequencing of perivascular adipose tissue (PVAT) depots (ta, aa, and mes), non-PVAT adipose tissues (BAT and WAT), and fecal samples. Samples were collected at two time points separated by one year: 2023 (rats 1–3) and 2024 (rats 4–6). We examined the full (unfiltered) dataset to retain all detectable taxa and minimize the exclusion of potentially biologically relevant low-abundance organisms. However, given the low-biomass nature of PVAT samples and the associated susceptibility to background signal and spurious detections, interpretation focused on taxa with relative abundance ≥1% to provide a more conservative and interpretable representation of microbial reads. Thus, the ≥1% threshold was applied as a reporting criterion rather than a detection filter, balancing sensitivity (through initial inclusion of the full dataset) with specificity (through focused interpretation of more robust signals). 

Analyses were conducted in R (version 4.3.2; R Foundation for Statistical Computing, Vienna, Austria) using the “vegan” package [31] and GraphPad Prism 10.4.1 Prism. Graphs were generated using GraphPad Prism 10.4.1 and R.

#### 2.3.1. Microbiome Community Dissimilarity Analysis and Statistics

To quantify differences in community composition and assess variability within and between sampling periods, Bray–Curtis dissimilarity was calculated from percent relative abundance values using the “vegdist” function. This metric captures compositional dissimilarity based on relative contributions of individual taxa and is commonly used for ecological and microbiome community comparisons [32].

Pairwise Bray–Curtis distances were computed across all samples to evaluate overall community dissimilarity, as well as within individual tissue types. Bray–Curtis distances were then explored using two approaches. First, we subjected the distance matrix to principal coordinates analysis (PCoA), using the command “cmdscale”, to visualize the dominant structures in the data set. Second, we used permutation-based PERMANOVA and Anderson’s dispersion test [33], using the commands “adonis2” and “betadisper”, respectively, to assess the significance of differences among groups. Due to limited sampling size, interactions were not formally tested (but can be assessed qualitatively in the ordinations). Using these methods, we evaluated the following questions: Do rats in different cohort years (2023, rats 1–3 vs. 2024, rats 4–6) have different microbiomes? Does the microbiome of PVATs differ from that of BAT and WAT? Is the microbiome of taPVAT more similar to BAT than the other adipose tissues, or are aaPVAT and mesPVAT more similar to WAT?

#### 2.3.2. Data Reporting

Microbial abundance data are reported as percentages of relative abundance for bacterial and viral species present at ≥1% of the total community. This threshold was applied to focus on biologically meaningful contributors to the microbial community and to minimize the influence of low-abundance microbial species and potential outliers that could obscure interpretation. The results are presented for each tissue type, and grouped data were used to depict relative abundance (%) across samples within each experimental cohort (1 and 2). The complete dataset, including microorganisms with relative abundances below 1% threshold, is deposited in the Supplementary Materials (File S4).

## 3. Results

**Figure 2:**
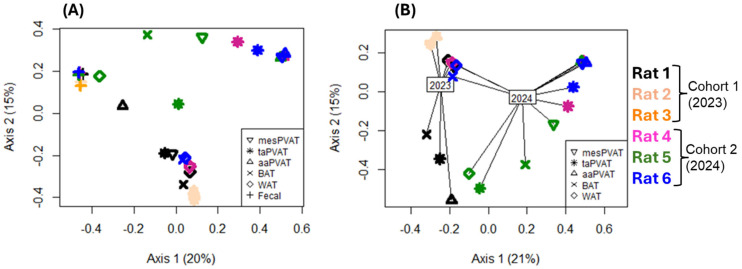
Bacterial community ordination using principal coordinates analysis based on Bray–Curtis distance. Rats from cohort 1 (2023) and cohort 2 (2024) are indicated by colors, and sample tissue types are indicated by symbol shape. Tissue types include thoracic aortic perivascular adipose tissue (taPVAT), abdominal aortic PVAT (aaPVAT), mesenteric PVAT (mesPVAT), subscapular brown adipose tissue (BAT), retroperitoneal white adipose tissue (WAT), and feces. Percents shown on axis labels indicate the proportion of variance in community composition that is summarized by that axis (i.e., the R^2^ of the axis). (**A**) All samples included. Fecal samples have a significantly different centroid and lower dispersion compared to other tissues (*p* < 0.01). (**B**) Only adipose tissues included (i.e., fecal samples excluded), with lines drawn from each sample point to the corresponding cohort centroid to illustrate the effect of year on the composition of detected bacteria (*p* < 0.01).

**Figure 3:**
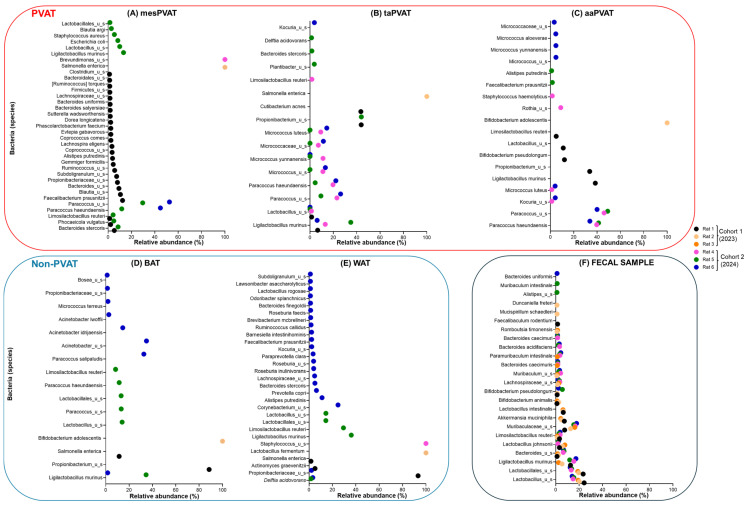
Relative abundance of diverse bacterial communities in PVAT and Non-PVAT across 2023 and 2024 cohorts. This scatter plot compares the relative abundance (%) of bacterial communities in perivascular adipose tissue (PVAT) and non-PVAT samples across cohort 1 (2023) and cohort 2 (2024). PVAT are shown in (**A**): mesenteric resistance artery (mesPVAT), (**B**): thoracic aorta (taPVAT), and (**C**): abdominal aorta (aaPVAT). Non-PVAT are shown in (**D**): brown adipose tissue (BAT) and (**E**): white adipose tissue (WAT). Positive control is shown in (**F**): fecal sample. The graphs display specific bacterial species (e.g., *Salmonella enterica*, *Paracoccus* spp., *Lactobacillus* spp.) as data points, with colors representing individual rats (Rat 1–6). The *x*-axis shows relative abundance, highlighting the temporal variability and diversity of bacterial communities, with PVAT exhibiting greater fluctuations than non-PVAT across the two cohorts.

**Figure 4:**
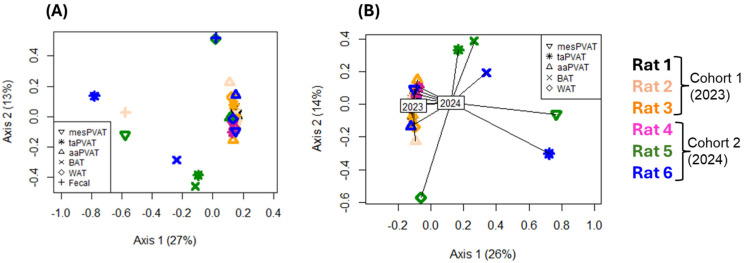
Viral community ordination using principal coordinates analysis based on Bray–Curtis distance. Rats from cohort 1 (2023) and cohort 2 (2024) are indicated by colors, and sample tissue types are indicated by symbol shape. Tissue types include thoracic aortic perivascular adipose tissue (taPVAT), abdominal aortic PVAT (aaPVAT), mesenteric PVAT (mesPVAT), subscapular brown adipose tissue (BAT), retroperitoneal white adipose tissue (WAT), and feces. Percents shown on axis labels indicate the proportion of variance in community composition that is summarized by that axis (i.e., the R^2^ of the axis). (**A**) All samples included. (**B**) Only adipose tissues included (i.e., fecal samples excluded), with lines drawn from each point to the corresponding cohort centroid to illustrate the significant effect of year on the dispersion (variability) and composition of detected viral species (*p* < 0.05).

**Figure 5:**
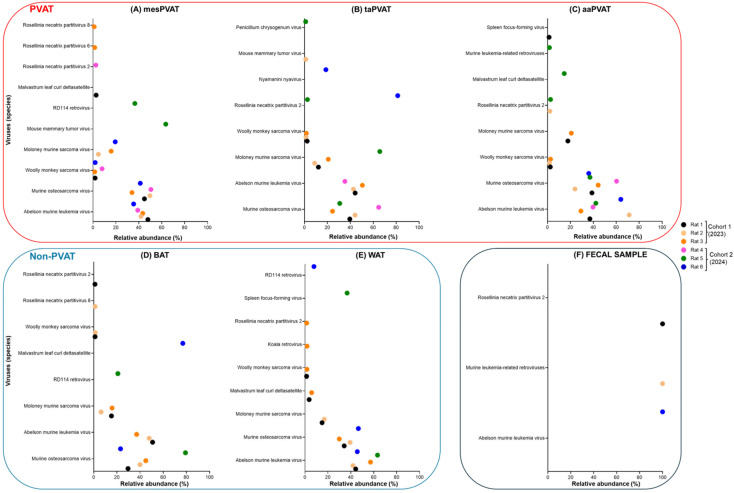
Viral species presence across PVAT and Non-PVAT in 2023 vs. 2024 cohorts. This scatter plot illustrates the relative abundance (%) of viral species in perivascular adipose tissue (PVAT) and non-PVAT samples across cohort 1 (2023) and cohort 2 (2024). PVAT are shown in (**A**) mesenteric resistance artery (mesPVAT), (**B**) thoracic aorta (taPVAT), and (**C**) abdominal aorta (aaPVAT). Non-PVAT are shown in (**D**) brown adipose tissue (BAT) and (**E**) white adipose tissue (WAT). Positive control is shown in (**F**) fecal sample. The graphs display key viral taxa (e.g., *Moloney murine sarcoma virus* and *Abelson murine leukemia virus*) as data points, with colors representing individual rats (Rat 1–6). The *x*-axis shows relative abundance, highlighting the consistent variability in viral species presence and abundance across both cohorts and tissue types.

In this section, we first present the results demonstrating cohort-specific inter-individual variability across samples, as shown in the bacterial ordination (Figure 2) and taxon abundances (Figure 3), stratified by date of collection. We subsequently report the viral ordination (Figure 4) and the relative abundances of taxa (Figure 5).

### 3.1. Microbiome Stratification into Cohorts

Whole-genome shotgun sequencing of DNA extracts from PVAT samples (thoracic aorta [ta], abdominal aorta [aa], and mesenteric resistance [mes] arteries), non-PVAT adipose samples that are brown (subscapular brown adipose tissue [BAT] and white (retroperitoneal adipose tissue WAT]), and fecal samples from six male Dahl SS rats revealed the presence of bacterial and viral gene sequences consistent with microbial DNA across all tissue types.

#### 3.1.1. Bacterial Communities’ Cohorts—Bray–Curtis Dissimilarity

The ordination of bacterial communities indicates several major patterns. First, when fecal samples are included in the ordination (Figure 2A), the first PCoA axis separates them from all other samples. In addition, all fecal microbiomes (from all rats and years) are clustered together (represented by the plus signs, Figure 2A) more than any adipose tissue type, indicating that bacterial species composition of adipose tissue is more variable than it is in fecal samples. Driven by the fecal samples, tissue types had significantly different centroids and dispersions (*p* < 0.01, Figure 2A). Second, the adipose tissues (PVAT and non-PVAT) from one rat (rat 2) are tightly clustered, driven by the very low diversity in these tissues (only one bacterial species detected in each). All other rats had highly variable bacterial species composition across adipose tissue types.

After removing fecal samples (Figure 2B) to better focus on relationships among adipose tissue communities, there is strong separation of communities by cohort, with samples from different years mostly clustering separately along PCoA axis 1. The time frame (one year between data collections) had a significant effect on bacterial composition centroids (*p* < 0.01), but not on dispersion. However, there is no clustering by adipose tissue type or caging history that is apparent from either ordination or permutation tests (*p* > 0.05).

Fecal samples showed lower dispersion (variation), as indicated by the clustering of fecal points (Figure 2A), but also had distinct bacterial compositions, as indicated by the centroids (points representing the means of multiple axes), and greater richness of bacterial communities. Centroid differences reflect shifts in overall community composition, whereas dispersion reflects within-group variability around that composition. In other words, the fecal sample showed much less variation (Figure 2A), while PVAT and non-PVAT showed similar variation (Figure 2B).

#### 3.1.2. Bacterial Relative Abundance and Variability of Species Across PVAT, Non-PVAT, and Fecal Samples

Below, we describe the species of bacteria with relative abundance greater than 1% in each sample (Figure 3A–F). Across all samples, whole-genome shotgun sequencing identified a heterogeneous bacterial composition, which varied by anatomical site, between cohorts, and among individual rats. 

Considering PVAT depots from cohorts 1 and 2, mesPVAT, taPVAT, and aaPVAT each harbored 35, 17, and 18 distinct bacterial species in total across all rats, respectively (Figure 3A–C). In non-PVAT samples, BAT showed 16 bacterial species, whereas WAT showed 30 (Figure 3D,E). Fecal samples had 23 different bacterial species (Figure 3F).

In both PVAT and non-PVAT groups, no common bacterial species with relative abundance > 1% were found across all six rats. Across adipose tissue depots, bacterial species exhibited partial, tissue-specific overlap among subsets of rats rather than universal presence, with shared taxa observed in some animals within the same cohort, in others across cohorts, and in others restricted to specific depots, highlighting pronounced inter-individual and site-dependent heterogeneity. In contrast, a core bacterial community was observed only in fecal samples, comprising seven shared species.

To further characterize this heterogeneity, bacterial overlap patterns were examined separately within non-PVAT adipose tissues (BAT and WAT) and across individual PVAT depots (mesenteric, thoracic, and abdominal), and the shared species were described within and between cohorts. In the following section, we describe the % of the most abundant bacterial taxa (among those with >1% relative abundance). The complete list of bacterial taxa shown in Figure 3A–F is provided in the Supplementary Materials (File S4).

***PVAT depots***

*mesPVAT*

The bacterial profile in mesPVAT exhibited pronounced inter-individual variability across rats and clear differences between cohorts (Figure 3A). In cohort 1 (2023), mesPVAT from rat 1 exhibited a diverse bacterial profile, with multiple taxa present at relative abundances above 1%. The most abundant species were *Faecalibacterium prausnitzii* (12.3%), *Blautia_u_s* (10.5%), *Bacteroides_u_s* (9.3%), and *Propionibacteriaceae* spp. (8.0%). Additional contributors are described in the Supplementary Materials (File S3). Rat 2 mesPVAT was dominated by a single taxon, *Salmonella enterica*, which accounted for 100.0% of the detected bacterial community, while rat 3 showed no bacterial species.

In cohort 2 (2024), mesPVAT samples showed fewer dominant taxa but marked variability among animals. Rat 4 mesPVAT was exclusively composed of *Brevundimonas_u_s* (100.0%). In rat 5, the community was more diverse, with *Paracoccus_u_s* (29.6%) as the dominant taxon. Rat 6 mesPVAT was dominated by *Paracoccus_u_s* (52.5%) and *Paracoccus haeundaensis* (44.8%), with no additional taxa exceeding 1%.

Only a limited number of bacterial taxa were shared across rats; most notably, mesPVAT samples collected one year apart (rats 1 and 5) shared three bacterial species: *Bacteroides stercoris, Phocaeicola vulgatus,* and *Limosilactobacillus reuteri*. 

*Paracoccus_u_s* and *Paracoccus haeundaensis* were commonly found in rats 5 and 6 (both in cohort 2).

*taPVAT*

taPVAT exhibited marked inter-individual variability, with distinct dominance patterns of bacterial communities across rats and between cohorts (Figure 3B), like the findings observed in mesPVAT. In cohort 1 (2023), taPVAT from rat 1 was dominated by two closely related taxa, *Propionibacterium_u_s* (43.8%) and *Cutibacterium acnes* (43.6%), together accounting for the majority of the detected bacterial community. In contrast, taPVAT from rat 2 was composed exclusively of *Salmonella enterica* (100.0%), whereas that from rat 3 contained no bacterial species. This is the same pattern as in mesPVAT—no bacterial species were found in rat 3, and only *Salmonella enterica* was observed in rat 2. Note that although these two rats are grouped in the same cohort (2023), their samples were collected three months apart.

In cohort 2 (2024), taPVAT samples displayed greater taxonomic diversity but remained highly variable among animals. Rat 4 exhibited a mixed community dominated by *Paracoccus_u_s* (23.1%) and *Paracoccus haeundaensis* (19.7%). Rat 6 taPVAT showed dominance of *Paracoccus_u_s* (26.2%) and *Paracoccus haeundaensis* (22.1%). Four common bacterial species were found in cohort 2: *Ligilactobacillus murinus*, *Lactobacillus_u_s*, *Paracoccus_u_s*, and *Paracoccus haeundaensis*. No common bacteria were identified among the rats in cohort 1. However, one rat in cohort 1 (rat 1) and all rats in cohort 2 shared a single bacterial species (*Ligilactobacillus murinus*).

*aaPVAT*

aaPVAT showed substantial inter-individual bacterial variability across rats and differences between cohorts (Figure 3C). In cohort 1 (2023), aaPVAT from rat 1 was characterized by five taxa: *Propionibacterium_u_s* (33.6%), *Ligilactobacillus murinus* (38.5%), *Bifidobacterium pseudolongum* (12.0%), *Lactobacillus_u_s* (11.0%), and *Limosilactobacillus reuteri* (5.0%). In contrast, rat 2 aaPVAT was dominated by a single taxon, *Bifidobacterium adolescentis* (100.0%), while rat 3 showed no bacterial species. Again, this pattern repeats as observed in mesPVAT and taPVAT.

In cohort 2 (2024), aaPVAT samples displayed distinct dominance patterns across animals. Rat 4 exhibited a community dominated by *Paracoccus_u_s* (46%) and *Paracoccus haeundaensis* (39.7%). In rat 6, the prevalence of *Paracoccus_u_s* (40.0%) and *Paracoccus haeundaensis* (33.8%) was observed. As shown in Figure 3C, in aaPVAT, taxonomic overlap across animals was restricted to cohort 2, with *Paracoccus haeundaensis* and *Paracoccus_u_s* shared among rats 4–6, whereas no shared taxa were observed among the cohort 1 animals. 

**non-PVAT**

*BAT*

Subscapular BAT showed pronounced inter-individual variability in cohort 2 compared to in cohort 1 (Figure 3D). In cohort 1 (2023), BAT from rat 1 was dominated by only two species, *Propionibacterium_u_s* (88.6%), with *Salmonella enterica* (11.4%) comprising the remaining bacterial community above the 1% threshold. Rat 2 BAT was exclusively composed of *Bifidobacterium adolescentis* (100.0%), whereas rat 3 showed no bacterial taxa exceeding 1% relative abundance.

In cohort 2 (2024), BAT samples exhibited more diverse bacterial profiles, although dominance patterns differed in rats 5 and 6. Rat 4 showed no taxa. In contrast, rat 5 contained multiple taxa above 1%, including *Ligilactobacillus murinus* (34.5%). Rat 6 displayed a distinct profile dominated by *Acinetobacter_u_s* (34.9%), followed by *Paracoccus salipaludis* (32.6%), and *Acinetobacter idrijaensis* (14.6%). Rats 5 and 6 shared only one bacterial species (*Ligilactobacillus murinus*); however, the percentage in rat 6 was very low (1.6%).

*WAT*

The retroperitoneal WAT we detected comprised 30 distinct species across rats and showed clear differences between cohorts (Figure 3E). In cohort 1 (2023), four species were identified, whereas in cohort 2, 26 species were identified. WAT from rat 1 presented three species, being dominated by *Propionibacteriaceae_u_s* (93.3%). In contrast, rat 2 WAT was composed exclusively of a single species (*Lactobacillus fermentum*), whereas rat 3 showed no bacteria. It is remarkable that this pattern is exactly that of BAT and all PVAT samples (i.e., no bacterial species in rat 3 and only one in rat 2).

In cohort 2 (2024), WAT samples exhibited distinct dominance patterns across animals, with the majority of species found in rat 6 (21 distinct bacterial species). In rat 6, WAT exhibited the highest taxonomic diversity, with multiple taxa present above 1%, including *Corynebacterium_u_s* (24.8%) and *Alistipes putredinis* (11.1%). Rat 5 displayed a moderate heterogeneous bacterial profile, with *Ligilactobacillus murinus* (36.1%) as the most abundant taxon, followed by *Limosilactobacillus reuteri* (29.5%), *Lactobacillus_u_s and Lactobacillales_u_s* (both 14.4%). Rat 4 WAT was exclusively dominated by *Staphylococcus_u_s* (100.0%).

**Fecal sample**

Fecal bacterial exhibited relatively consistent profiles across rats, with multiple taxa contributing moderate relative abundances in both cohorts (Figure 3F). This is markedly important in providing support for our experimental protocol and ensuring confidence in data derived from rat 3, in which no adipose tissue microbiome was observed.

Fecal microbiota displayed relatively consistent community structures across both cohorts, dominated by *Lactobacillus_u_s*, *Lactobacillales_u_s*, *Muribaculaceae_u_s*, and *Ligilactobacillus murinus* (ranging from 2.4–24.2%), with cohort-specific differences observed in lower-abundance taxa.

In cohort 1 (2023), fecal samples from rats 1, 2, and 3 were dominated *by Lactobacillus_u_s* (24.2, 20, 19.1%) *and Lactobacillales_u_s* (23.4, 19.6, 18.8%), together accounting for a substantial proportion of the bacterial community. Bacteria shared across all three rats included *Muribaculaceae_u_s*, *Ligilactobacillus murinus*, *Akkermansia muciniphila*, *Lactobacillus intestinalis*, *Lactobacillus johnsonii*, *Limosilactobacillus reuteri*, and *Bacteroides_u_s* and *Bifidobacterium animalis*.

In cohort 2 (2024), fecal profiles for rats 4, 5, and 6 were similarly structured, with *Lactobacillus_u_s* (14.8, 15.4, 14.0%) and *Lactobacillales_u_s* (13.2, 13.5, 12.3%). *Muribaculaceae_u_s* again represented a major component (16.7, 15.4, 17.8%), alongside *Ligilactobacillus murinus* (15.5, 11.9, 17.2%). In contrast to cohort 1, *Akkermansia muciniphila* and *Lactobacillus intestinalis* were not detected at >1% in fecal samples from cohort 2.

Across fecal samples, seven bacterial taxa were consistently detected in all rats from both cohorts at relative abundances exceeding 1%, including *Lactobacillus_u_s*, *Lactobacillales_u_s*, *Ligilactobacillus murinus*, *Bacteroides_u_s*, *Lactobacillus johnsonii*, *Limosilactobacillus reuteri*, and *Muribaculaceae_u_s*, each contributing moderate relative abundances across animals.

Although fecal samples exhibited the greatest number of bacterial taxa shared across all rats and both cohorts—a pattern not observed in PVAT or non-PVAT adipose tissues—the maximum relative abundance of any single fecal taxon was modest, reaching 24.2%, which was substantially lower than the dominant taxa observed in PVAT depots (mesPVAT, taPVAT, aaPVAT) and non-PVAT adipose tissues (BAT and WAT).

#### 3.1.3. Viral Communities’ Cohorts—Bray–Curtis Dissimilarity

Ordination of viral communities did not reveal clustering by any tissue type, including feces, compared to PVAT and non-PVAT adipose tissues (Figure 4A; *p* > 0.05).

Fecal viral communities were characterized by extreme inter-individual specificity, with dominance of a single viral taxon when present and complete absence of ≥1% viruses in several animals. Notably, no viral species ≥1% of relative abundance were detected in fecal samples from rat 4, consistent with the absence observed in BAT and WAT from the same animal.

Interestingly, no viral species were detected in BAT, WAT, and fecal samples from rat 4.

Rats in cohort 2 were housed together and exhibited a highly diverse viral profile across samples. After removing fecal samples, the strong impact of cohort became apparent (Figure 4B). Year had a significant effect on both viral composition centroids and dispersion (*p* < 0.01), with 2024 samples showing much greater composition variance than 2023 samples. Viral species relative-abundance profiles differed in rats 1–3, exhibiting more consistent tissue-specific compositions than rats 4–6, which, overall, showed greater inter- and intra-individual variability (Figure 4A,B).

#### 3.1.4. Viral Relative Abundance and Variability of Species Across PVAT, Non-PVAT, and Fecal Samples

Considering PVAT depots from cohorts 1 and 2, mesPVAT, taPVAT, and aaPVAT each harbored 17, 15, and 13 distinct virus species, respectively, across all rats sampled (Figure 5A–C). Among PVAT depots, mesPVAT, aaPVAT, and taPVAT shared eight virus species (*Abelson murine leukemia virus*, *Moloney murine sarcoma virus*, *Murine leukemia-related retroviruses*, *Murine osteosarcoma virus*, *Penicillium chrysogenum virus*, *RD114 retrovirus, Rosellinia necatrix partitivirus 2*, and *Woolly monkey sarcoma virus*). Detected viral sequences were predominantly annotated as murine retroviruses. 

In non-PVAT samples, BAT and WAT showed a similar amount of species (15 and 14, respectively) (Figure 5D,E), sharing nine virus species (*Abelson murine leukemia virus*, *Malvastrum leaf curl deltasatellite*, *Feline leukemia virus*, *Moloney murine sarcoma virus*, *Murine osteosarcoma virus*, *Penicillium chrysogenum virus*, *RD114 retrovirus*, *Rosellinia necatrix partitivirus 2* and *Woolly monkey sarcoma virus*).

Across adipose compartments, there are overlapping viral profiles between vascular-associated and non-vascular adipose compartments. PVAT depots (mesPVAT, aaPVAT, and taPVAT) and non-PVAT tissues (BAT and WAT) shared seven virus species (*Abelson murine leukemia virus*, *Malvastrum leaf curl deltasatellite*, *Feline leukemia virus*, *Moloney murine sarcoma virus*, *Murine osteosarcoma virus*, *Rosellinia necatrix partitivirus 2*, and *Woolly monkey sarcoma virus*). In contrast, fecal samples exhibited a profoundly limited viral repertoire, with only three virus species detected, all of which were also present in non-PVAT adipose tissues and overlapped with PVAT depots (Figure 5F).

Below, we describe the most abundant viral taxa across tissues, stratified by cohort, as depicted in Figure 5A–F. The complete list of viral taxa with relative abundance >1% is provided in Supplementary File S3.

**PVAT Depots**

*mesPVAT*

Viral sequence detected in mesPVAT exhibited pronounced inter-individual variability across rats and differences between cohorts (Figure 5A). In mesPVAT, all rats from cohort 1 (rats 1–3) and two rats from cohort 2 (rats 4 and 6) exceeded the 1% relative abundance threshold for *Abelson murine leukemia virus* and *Murine osteosarcoma virus.*

In cohort 1 (2023), mesPVAT from rats 1–3 was dominated by *Abelson murine leukemia virus* (47.9, 41.9, 43.1%) and *Murine osteosarcoma virus* (44.9, 59.7, 34.1%), which together accounted for the majority of viral relative abundance in all three animals.

In cohort 2 (2024), mesPVAT viral profiles differed markedly across animals. Rat 4 showed dominance by *Murine osteosarcoma virus* (50.5%) and *Abelson murine leukemia virus* (39.1%), with *Woolly monkey sarcoma virus* accounting for an additional 7.9%. In contrast, rat 5 exhibited a distinct viral profile dominated by *Mouse mammary tumor virus* (63.5%) and *RD114 retrovirus* (36.5%), with no other viral species exceeding the 1% threshold. Rat 6 mesPVAT again showed high relative abundances of *Abelson murine leukemia virus* (35.5%) and *Murine osteosarcoma virus* (41.3%), with *Moloney murine sarcoma virus* (19.4%) and *Woolly monkey sarcoma virus* (1.9%) also detected.

*taPVAT*

In PVAT from the thoracic aorta, perivascular adipose tissue (taPVAT), we found marked inter-individual variability across rats and differences between cohorts (Figure 5B). In cohort 1 (2023), taPVAT from rats 1–3 was dominated by *Abelson murine leukemia virus* (44.2, 42.6, 50.5%) and *Murine osteosarcoma virus* (39.5, 44.0, 24.4%), which together accounted for the majority of viral relative abundance in all three animals. *Moloney murine sarcoma virus* was also consistently detected in cohort 1, contributing 12.1% in rat 1, 8.8% in rat 2, and 20.7% in rat 3.

In cohort 2 (2024), taPVAT viral profiles diverged substantially between animals. Rat 4 exhibited strong dominance of *Murine osteosarcoma virus* (64.7%) and *Abelson murine leukemia virus* (35.3%), with no additional viral species exceeding 1%. In contrast, rat 5 showed a distinct profile dominated by *Moloney murine sarcoma virus* (65.7%) and *Murine osteosarcoma virus* (30.8%), accompanied by *Rosellinia necatrix partitivirus* 2 (2.5%). Rat 6 taPVAT was characterized by a high relative abundance of *Rosellinia necatrix partitivirus* 2 (81.4%) and *Nyamanini nyavirus* (18.6%), with no other viral species detected above the 1% threshold. taPVAT viral communities were dominated by a small number of viral species, with *Abelson murine leukemia virus* and *Murine osteosarcoma virus* prevalent in cohort 1, whereas in cohort 2, taPVAT viral profiles differed markedly between animals, with rat 4 dominated by murine retroviruses, rat 5 dominated by *Moloney murine sarcoma virus*, and rat 6 dominated by *Rosellinia necatrix partitivirus* 2. *Murine osteosarcoma virus* was detected in all rats from both cohorts except rat 6 (cohort 2), indicating its widespread presence across animals despite inter-individual variability in viral dominance patterns.

*aaPVAT*

Viral communities detected in abdominal aorta perivascular adipose tissue (aaPVAT) were dominated by a small number of viral species across rats, with differences in relative abundance observed between cohorts (Figure 5C). In cohort 1 (2023), aaPVAT from rats 1–3 was consistently dominated by *Abelson murine leukemia virus* (37.0, 71.3, 29.1%) and *Murine osteosarcoma virus* (38.8, 23.9, 44.2%). In cohort 2 (2024), aaPVAT viral profiles remained dominated by the same two retroviruses but with notable inter-individual variability. Rats 4–6 showed high relative abundances of *Murine osteosarcoma virus* (60.3, 37.1, 36.0%) and *Abelson murine leukemia virus* (39.7, 42.2, 64.0%). Remarkably, in aaPVAT, two viral communities were consistently dominant across both cohorts: *Abelson murine leukemia virus* and *Murine osteosarcoma virus*, with reasonably high relative abundances (>24%).

**Non-PVAT**

*BAT*

Viral communities detected in BAT displayed marked inter-individual variability and distinct patterns between cohorts (Figure 5D). In cohort 1 (2023), BAT samples from rats 1–3 were dominated by *Abelson murine leukemia virus* (50.8, 47.9, 37.0%), *Murine osteosarcoma virus* (29.6, 40.0, 44.9%), and Moloney murine sarcoma virus (15.2, 6.3, 15.8%).

In cohort 2 (2024), BAT viral profiles differed substantially across animals. Rat 4 showed no viral species. In contrast, rat 5 BAT was dominated by *Murine osteosarcoma virus* (79.2%), with *RD114 retrovirus* contributing an additional 20.8%. Rat 6 exhibited a distinct profile characterized by strong dominance of *Malvastrum leaf curl deltasatellite* (76.9%), accompanied by *Murine osteosarcoma virus* (23.1%). No other viral taxa exceeded the 1% threshold in cohort 2 BAT samples. Except for rat 4 (cohort 2), the Murine osteosarcoma virus was detected in BAT samples from all rats across both cohorts.

*WAT*

Viral communities detected in WAT (retroperitoneal) showed substantial inter-individual variability across rats and clear differences between cohorts (Figure 5E). In cohort 1 (2023), WAT samples from rats 1–3 were consistently dominated by *Abelson murine leukemia virus* (44.2, 41.9, 57.1%) and *Murine osteosarcoma virus* (34.2, 39.3, 29.9%). 

In cohort 2 (2024), WAT viral profiles diverged markedly between animals. Rat 4 showed no viral species exceeding the 1% relative abundance threshold. In contrast, rat 5 WAT was dominated by Abelson murine leukemia virus (63.3%), accompanied by *Spleen focus-forming virus* (36.7%). Rat 6 displayed a distinct profile characterized by high relative abundances of *Abelson murine leukemia virus* (45.6%) and *Murine osteosarcoma virus* (46.5%)

**Fecal Samples**

Fecal viral communities were sparse and highly individualized, with single-virus dominance observed in isolated animals and no viral species shared among rats or cohorts (Figure 5F). In fecal samples, only three viral species were detected. These were limited to three animals: rats 1 and 2 (both Cohort 1, 2023), which harbored *Rosellinia necatrix partitivirus 2* and *Murine leukemia-related retroviruses*, respectively, and rat 6 (Cohort 2, 2024), which was the only one harboring *Abelson murine leukemia virus*. No viral species were detected in fecal samples from any of the other animals, indicating that detectable fecal viral profiles were sparse and highly individualized across cohorts and independent of collection time points (Figure 5F). Notably, among the three viruses identified in fecal samples, *Rosellinia necatrix partitivirus* 2 and *Abelson murine leukemia virus* were detected above the 1% relative abundance threshold in both all PVAT (Figure 5A–C) and non-PVAT (Figure 5D,E) samples. In contrast, *Murine leukemia-related retroviruses* were found only in aaPVAT (Figure 5C).

In contrast to bacterial communities, no viral taxa were shared across all tissue types when fecal samples were included. However, several murine retroviruses—most notably *Abelson murine leukemia virus* and *Murine osteosarcoma virus*—were consistently detected across all PVAT depots and non-PVAT adipose tissues, with fecal viral profiles showing sparse, animal-specific dominance patterns.

## 4. Discussion

*Bacterial Community Patterns Across Depots*

Across PVAT, non-PVAT adipose tissues, and feces, only three bacterial taxa exceeded the 1% relative abundance threshold in all tissue types: *Lactobacillus (unclassified species), Ligilactobacillus murinus*, and *Limosilactobacillus reuteri*. Their relative abundances varied substantially by tissue and by animal, indicating that cross-tissue presence does not imply uniform representation. In contrast to adipose tissues, fecal samples exhibited a broader shared core across animals and cohorts.

These findings suggest that adipose-associated bacterial community—particularly within PVAT—are not simple reflections of fecal microbiota but instead represent selective, tissue-associated profiles characterized by limited overlap and substantial inter-animal variability. Importantly, the wide range of relative abundances observed for those shared taxa across tissues further emphasizes that cross-tissue presence does not imply uniform dominance. This underscores the need to consider both prevalence and relative abundance when interpreting microbial composition across anatomical niches.

The absence of a consistent “core” bacterial microbiome across PVAT samples, together with the detection of low or absent bacterial signal in some specimens, suggests substantial variability in microbial profiles. Several explanations should be considered when interpreting these findings. First, PVAT represents a low-biomass environment, and microbial DNA may be present at or near the limits of detection, leading to stochastic taxon identification across samples. Second, inter-individual variability may contribute to heterogeneous microbiomes, particularly in small cohorts. Microbial signals could arise from the translocation of microbial components or cell-free DNA from adjacent tissues, the circulation, or the gastrointestinal tract. Finally, technical factors are discussed in the limitations. Accordingly, our findings are best interpreted as evidence of detectable microbiomes in PVAT, warranting further investigation in larger, rigorously controlled studies.

*Viral Community Structure and Restricted Overlap*

Compared to bacterial profiles, viral profiles exhibited a markedly more restricted overlap across tissues, with no viral taxa detected above the 1% relative abundance threshold across all sampled tissue types when fecal samples were included (Figure 5A–F).

Seven viral taxa were detected in both PVAT and non-PVAT depots, including multiple murine retrovirus reference genomes (e.g., *Abelson murine leukemia virus*, *Moloney murine sarcoma virus*, *Woolly monkey sarcoma virus*, and *Murine osteosarcoma virus*), as well as non-mammalian viral references.

Viral profiles were characterized by dominance of a limited number of taxa and pronounced variability in relative abundance across animals and cohorts, whereas fecal viral communities remained sparse and highly individualized. These patterns suggest that viral detection in adipose tissues reflects restricted and heterogeneous viral profiles rather than a uniform or stable virome, with inter-individual variability largely driven by disproportionate dominance of specific taxa within individual samples.

Interpretation of these findings requires caution, particularly in low-biomass metagenomic datasets. Because endogenous retroviral elements are pervasive within mammalian genomes and can be transcribed, and because low-biomass metagenomic datasets are susceptible to reagent and laboratory background contamination, shared retroviral detections should be interpreted as overlapping sequence signatures rather than definitive evidence of compartment-specific productive infection [34].

Detected retroviral sequences may originate from endogenous retroviral elements embedded within the host genome rather than exogenous viral populations. In addition, sequence homology between host-derived retroelements and reference viral databases may contribute to taxonomic misclassification, especially in the absence of stringent host read depletion or virome-specific analytical pipelines.

Taken together, these considerations indicate that the observed viral profiles likely reflect a combination of biological signal and technical or annotation-related factors. Therefore, these findings should not be interpreted as definitive evidence of an active or resident PVAT virome but rather as preliminary observations requiring further validation using approaches specifically designed to resolve host–virus sequence ambiguity.

*Do mes, aa, and taPVAT samples collected one year apart present different microbiomes?*

In bacterial analyses, after removal of fecal samples, the adipose depots cohort year was associated with a significant shift in community centroids, with no differences in dispersion (Figure 2B), indicating consistent compositional restructuring between 2023 and 2024. In contrast, viral communities demonstrated both centroid shifts and increased dispersion in 2024 (Figure 4B), suggesting greater inter- and intra-individual heterogeneity in the virome over time relative to the bacteriome. Within individual depots, aaPVAT exhibited relatively consistent cohort-specific patterns; however, this did not translate into overall depot-driven clustering across the dataset. Bacterial communities in cohort 2 aaPVAT were characterized by reproducible dominance of *Paracoccus* spp. (Figure 3C), while viral communities in cohort 1 aaPVAT were uniformly dominated by murine retroviruses (Figure 5C). In contrast, cohort 1 bacterial profiles in PVAT were highly heterogeneous, whereas viral profiles in the same cohort were comparatively conserved.

*Does PVAT differ from BAT and WAT?*

The data we share do not support PVATs (white or brown) being different in their microbiome family from WAT and BAT, respectively. Although for different reasons, neither viral nor bacterial PVAT presented differences from non-PVAT depots. In bacterial species, the absence of separation, as evidenced by the lack of clustering by adipose tissue type, indicated no significant differences in bacterial community structure. Ordination of viral communities did not reveal clustering by any tissue type, indicating no separation between PVAT, BAT and WAT, nor between fecal and adipose depots, unlike bacteria.

*Is taPVAT more similar to BAT? Or is aaPVAT/mesPVAT more similar to WAT?*

It is known that the PVAT phenotype is dependent on its anatomical location, with taPVAT exhibiting a brown PVAT signature, while aaPVAT shows a WAT-like phenotype [35]. Would the microbiome of taPVAT be more similar to BAT than to the other adipose tissues? Would aaPVAT and mesPVAT be more similar to WAT? Evidence does not support structured similarity in either viral or bacterial communities. 

*Environmental and Housing Considerations*

The environmental context may have contributed to the observed cohort stratification. Rats in cohort 2 were group-housed together, whereas cohort 1 included both single- and co-housed animals. Housing conditions are known to influence microbial exchange in rodents [36] and could partially contribute to between-cohort structuring. However, all animals were maintained on the same chow formulation, and similarities observed across individual animals from different cohorts argue against a purely cage-driven effect. Although the present study was not powered to formally disentangle housing from temporal effects, the consistent cohort-level separation across both bacterial and viral datasets suggests that year-associated environmental or colony-level factors likely exert stronger influence than adipose depot identity in this model.

*PVAT as a Resident Microbiome*

Across both bacterial and viral communities, PVAT and non-PVAT exhibited pronounced cohort-specific signatures, whereas non-PVAT adipose depots showed greater inter-animal variability. Overall, the temporal cohort emerged as the dominant organizing factor of microbiome structure, whereas adipose depot identity did not significantly structure community composition.

Our detection of diverse bacterial genera, including *Paracoccus*, *Salmonella*, *Lactobacillus*, and *Ligilactobacillus*, in PVAT aligns with emerging reports on the microbiota in adipose depots [19,37,38]. Prior work has largely focused on indirect gut–PVAT interactions mediated by circulating metabolites (e.g., TMAO [39,40] in models of metabolic disease, focused on the indirect effects of gut microbiota on PVAT function rather than the resident communities [41]. Our findings suggest that adipose tissues themselves harbor detectable microbial signatures beyond the gut, though their biological relevance remains to be determined.

*Can we say there is a PVAT-resident microbiome?*

Microbial sequences are detectable across PVAT and non-PVAT depots and in feces in Dahl SS rats, but the structure and overlap of these communities differ between bacteria and viruses and across adipose tissue depots, and the time of collection influences these patterns. Given the low-biomass nature of PVAT samples and the associated susceptibility to background signal and spurious detections, interpretation focused on taxa with relative abundance ≥1% to provide a more conservative and interpretable representation of microbial composition. PVAT contains a stromal vascular fraction enriched in immune cells, including macrophages and T lymphocytes [13,15,16]. PVAT is also known to modulate vascular tone [3,14,42], and microbial components could amplify this via immunomodulation or metabolite production [1,43]. The detection of microbiomes within PVAT raises the possibility that tissue-associated microbial components may interact with local immune and vascular pathways. Our work provides a foundational, descriptive framework for future studies aimed at exploring the biological relevance of tissue-associated microbiomes in cardiovascular and metabolic contexts while underscoring the need for careful, compartment-specific interpretation.

*Limitations*

This study has several important limitations that should be considered when interpreting the findings. First and most importantly, the small sample size (*n* = 6 rats; 3 per cohort) substantially limits statistical power and precludes robust inferential analysis. As such, this work should be considered exploratory and hypothesis-generating rather than definitive. In particular, observed differences between cohorts (2023 vs. 2024) may reflect inter-individual variability or uncontrolled environmental factors rather than true biological or temporal effects. Accordingly, statistical comparisons and interpretations of cohort-specific patterns should be approached with caution due to the increased risk of both type I and type II errors.

Cohort-specific differences also raise considerations regarding reproducibility. While these findings are transparently reported, they may be influenced by external variables such as environmental conditions, sample handling, storage, or batch effects inherent to sequencing workflows. These factors cannot be fully excluded and may contribute to the observed variability.

In addition, perivascular adipose tissue (PVAT) falls within the category of low-biomass microbiome, which is inherently susceptible to exogenous DNA contamination from laboratory reagents, DNA extraction kits, the laboratory environment, and sample handling. To mitigate these risks, stringent aseptic techniques were employed throughout sample collection and processing, including dissection in a laminar flow hood, use of autoclaved and single-use instruments, and the use of freshly opened sterile consumables. DNA extraction was performed using the QIAGEN DNeasy PowerSoil Pro Kit (Germantown, MD, USA), and library preparation was performed using the Illumina Nextera XT DNA Library Preparation Kit (San Diego, CA, USA) with unique dual indexing to reduce index cross-contamination.

Despite these precautions, contamination cannot be entirely excluded, as recognized in low-biomass microbiome research. Therefore, some detected taxa—particularly those at low relative abundance—may reflect background or reagent-associated DNA rather than true biological signals. This is especially relevant when interpreting variability across samples or cohorts. In addition, single-species dominance (where a species was found at 100% relative abundance) may reflect limitations inherent to low-biomass sequencing, including potential artifacts or misclassification by the bioinformatics pipeline. Multiple bioinformatics pipelines and complementary validation approaches (e.g., targeted sequencing or qPCR) will be important for confirming taxonomic assignments.

Furthermore, this study relies on relative abundance metrics derived from whole-genome sequencing without absolute quantification or functional validation, limiting insights into microbial activity and biological relevance. Finally, the use of the Dahl salt-sensitive rat model, while appropriate for vascular studies, may limit generalizability to human physiology, where PVAT composition and environmental exposures differ.

Taken together, these limitations underscore the need for future studies with larger cohorts, and complementary functional analyses to validate and extend these preliminary observations.

## 5. Conclusions

PVAT from the Dahl SS rat harbors distinct bacteria and viruses, with bacterial composition showing cohort-specific temporal variability, unlike stable viral profiles. Non-PVAT and fecal samples exhibited lower overall variability than PVAT samples. Our analyses did not identify differences in microbiome composition between PVAT surrounding conductance vessels (thoracic and abdominal aorta) and resistance vessels (mesenteric arteries), nor significant clustering by PVAT depot in either bacterial or viral communities. Bray–Curtis ordination indicated that cohort (year of collection) and inter-individual variability had a stronger influence on community composition than anatomical location. 

Temporal and environmental variables appear to exceed anatomical depot identity in structuring adipose-associated microbial communities. The greater dispersion observed in viral communities further suggests that the adipose virome may be more dynamically variable across sampling periods than the bacterial microbiome, although mechanistic drivers cannot be inferred from the present data. 

This study paves the way for investigating PVAT microbial roles in vascular homeostasis, immune modulation, and potential pharmacological targets for microbiome-based interventions in cardiovascular dysfunction. Future studies should incorporate larger cohorts, absolute quantification approaches, orthogonal validation methods, human tissues, and disease models. Interventional strategies such as microbiome modulation, transplantation paradigms, and targeted pharmacological study designs are necessary to determine whether tissue-associated microbes exert causal effects on vascular homeostasis or immune regulation. 

## Data Availability

The original data presented in this study are openly available as Supplementary Materials [Excel files named: File S1. TISSUE COLLECTION, File S2. Table (Sequencing Depth), File S3. Table (Relative Abundance Higher than 1%), File S4. Table_BACTERIA-TOTAL and VIRUSES-TOTAL].

## Supplementary Materials

The following supporting information can be downloaded at: https://www.mdpi.com/article/10.3390/life16040609/s1, File S1. TISSUE COLLECTION.doc, File S2. Table Sequencing Depth.exe, File S3. Table (Relative Abundance Higher than 1%).exe, File S4. Table_BACTERIA-TOTAL and VIRUSES-TOTAL.exe.
