# Supplementary material for: Presence and Variability of the Microbiome in Perivascular Adipose Tissue: A Whole-Genome Sequencing Study in Dahl SS Rats
Source: Life (Basel). 2026 Apr 7;16(4):609. doi: 10.3390/life16040609 (PMC13118091; doi:10.3390/life16040609)
Supplement: Supplementary file 1 [file life-16-00609-s001.zip › S1.TISSUE COLLECTION.pdf]

Supplementary Material – Sample extraction and preparation

Previous preparation (one or 2 days before sacrificing the animals):

1) Labeling the sterile microcentrifuge tubes (e.g., *"rat number" / sample type/ 'Lab's reference' / date: MM-DD-YY*).
1) Weigh the empty microcentrifuge tubes, keeping them sterile in individual plastic bags, and record values.
2) Autoclave surgical tools: one set for each animal, plus one extra set if needed. Each set included: two fine forceps, one large forceps, two small scissors, and two large scissors.

On the day of the experiment:

1) Preparing the surgical area and the animal:
   a. Physiological salt solution (PSS), kept at room temperature (volume depends on the number of animals). PSS [mM]: NaCl 130; KCl 4.7; KH2PO4 1.18; MgSO4·7H2O 1.17; NaHCO3 14.8; dextrose 5.5; CaNa2EDTA 0.03, CaCl2 1.6 (pH 7.2)]
   b. A container with dry ice.
   c. A container with regular ice.
   d. A container with liquid nitrogen.
   e. Clean the bench with a solution containing 2.0% sodium hypochlorite.
   f. Place the 1st set of surgical tools in the disinfected area.
   g. Place ethanol-washed beakers in the disinfected area.
   h. Place a beaker with approx. 500 mL of PSS in regular ice and a beaker with 70% ethanol to clean tools between tissues during dissections. (During the dissections, to avoid the accumulation of debris, surgical tools were constantly cleaned with ethanol and then rinsed with PSS).
   i. Prepare the rack with the labeled and sterile tubes.
   j. Anesthetize the animal (sodium pentobarbital (60-80 mg/kg, IP). The absence of the paw pinch and eye-blink reflexes confirmed deep anesthesia. Before complete euthanasia, areas from which samples were collected were shaved: thorax, chest, neck, abdomen, and the animals were weighed. A gauze soaked in 70% alcohol was used to remove excess hair and maintain aseptic conditions.
   k. Decapitation and exsanguination were performed prior to tissue removal, and tissues were removed for one of the following protocols.

2) Removing/Isolating the samples:
   a. All samples were immediately stored in liquid nitrogen. However, while pieces of samples from the same area are being collected, the tube can be kept in dry ice):
   b. Tissues were removed in the following order: BAT, WAT, taPVAT, aaPVAT, mesPVAT, and feces. BAT, WAT, taPVAT, and feces were immediately placed in the appropriate microcentrifuge-plate centrifuged tubes and stored in liquid

nitrogen. aaPVAT and mesPVAT were dissected from vessels in a dish containing PSS, then in the appropriate microcentrifuge-plate centrifuged tubes and stored in liquid nitrogen.

c. Following tissue collection, tubes were individually removed from the liquid nitrogen, dried with a laboratory paper towel, weighed, the weight recorded, and the tube placed back in liquid nitrogen (less than 30 seconds total time).

3) Storing and preparing the sample to ship to CosmosID:

a. Sample tubes were firmly sealed with a parafilm membrane to prevent them from opening during transportation.
b. Tube(s) were placed in a freezer box and then a cardboard box as secondary containment to protect the plastic rack tube from breaking during transport.
c. Samples were sent in a refrigerated, thermostable container, packed with 5 lb. (2.3 kg) dry ice for each 24h in transit. For example, if the shipment would take up to 2 days, a minimum of 10 lbs of dry ice would be needed.
d. Samples were shipped to CosmosID, allowing 1-2 days for shipping, with overnight recommended. Courier-specific guidelines and standards for shipping non-hazardous research samples were followed.
